# Authors’ Response to Peer Reviews of “COVID-19 Infection and Symptoms Among Emergency Medicine Residents and Fellows in an Urban Academic Hospital Setting: Cross-sectional Questionnaire Study”

**DOI:** 10.2196/36198

**Published:** 2022-01-27

**Authors:** Stacey Frisch, Sarah Jones, James Willis, Richard Sinert

**Affiliations:** 1 Department of Emergency Medicine SUNY Downstate Health Sciences University Brooklyn, NY United States; 2 Department of Emergency Medicine Jackson Health System Miami, FL United States

**Keywords:** COVID-19, emergency medicine, housestaff wellness, medical education, training, frontline health care workers, frontline, personal protective equipment, pandemic, infectious disease, emergency

*This is the authors’ response to peer-review reports for“COVID-19 Infection and Symptoms Among Emergency Medicine Residents and Fellows in an Urban Academic Hospital Setting: Cross-sectional Questionnaire Study.”*

The authors of the manuscript [1] are grateful to the editor and reviewers [2,3] for their invaluable input and feedback.

## Round 1

### Reviewer S [2]

#### Major Comments

1. We have added to the Data Analysis section of our paper in response to this reviewer’s comments. We chose the Fisher exact test instead of the Monte Carlo Simulation because we thought the readers would be more familiar with the Fisher exact test.

#### Minor Comments

2. We chose these variables based on our predetermined survey questions.

3. We investigated other statistical analyses by combining other variables but still found the sample size too small to make for a sound analysis.

4. This was a rounding error and should read .049. It has now been corrected. Thank you for pointing this out.

Data analysis: Survey responses were tabulated and compiled into a table format with ranges. Frequency data were reported as percentages with 95% CI. Group comparisons were analyzed by either by chi-square or the Fisher exact test if the sample size requirements for chi-square were violated (the value of the cell expected should be 5 or more in at least 80% of the cells, and no cell should have an expected value of less than 1; Berwick et al [4]). Alpha set as .05 and all tests were 2-tailed. SPSS Statistics for Windows (version 23.0, IBM Corp) was used.

### Reviewer BZ [3]

1,2. We have changed our wording to document an acceptable rate of survey completion. We also added the number of survey question items to the Methods section, as this is a known factor for survey noncompletion.

3,4. We have defined COVID-19 and SARS-CoV-2 the first time they are mentioned in the introduction of the paper. We have reread and edited our paper for punctuation and grammatical errors, and we used Grammarly software to assist with this process as well.

5. Please see our above comments on data analysis.

6. As the subjects of the study were physicians in training, the governing body dictating protocol was the Accreditation Council for Graduate Medical Education, which at the time and currently deferred to local institutional protocols apart from maintaining the need for supervision and providing adequate protective equipment. At the time this study was conducted, institutional protocol changed often so there was no uniform process for testing or quarantine. We have added this point to our discussion and feel discussing other countries’ protocols would not be appropriate for this publication.

7. We have corrected the use of SARS-CoV-2 and COVID-19 throughout our manuscript.

8. We listed the polymerase chain reaction (PCR) and antibody testing methods in our Methods section under study protocol, with references for each. We also cited the suggested article with a comment on clinical versus laboratory diagnosis in our Discussion section.

9. We have reviewed these articles and cited them in our Discussion section, along with commentary on their prevalence and presentation of COVID-19 compared to ours.

## Round 2 Review

Thank you for your comments.

1. We have changed this to include data up to October 2021 and labeled them as such.

2. We have added a second table (the new Table 2) with this data and added a paragraph to the manuscript with a summary of the data and table.

3. Since we found no trend in the univariate analysis of postgraduate year, clinical hours, or number of patients with COVID-19 treated, we decided that a receiver operator characteristic curve analysis or logistic regression was not appropriate to predict antibody positivity among our respondents.

## Round 3 Review

Thanks for your review. We have added the letter from the SUNY Downstate Institutional Review Board (IRB) confirming that this study is exempt from IRB approval to the Multimedia Appendices section.

## Abbreviations

IRB: instituional review board
PCR: polymerase chain reaction
